# Functionalization of Neutral Polypropylene by Using Low Pressure Plasma Treatment: Effects on Surface Characteristics and Adhesion Properties

**DOI:** 10.3390/polym11020202

**Published:** 2019-01-24

**Authors:** Chiara Mandolfino, Enrico Lertora, Carla Gambaro, Marco Pizzorni

**Affiliations:** Department of Mechanical Engineering, University of Genoa, Polytechnic School, Via All’ Opera Pia 15, 16145 Genoa, Italy; e.lertora@unige.it (E.L.); gambaro@dime.unige.it (C.G.); marco.pizzorni@dime.unige.it (M.P.)

**Keywords:** low pressure plasma, adhesive bonding, polypropylene, surface energy, AFM, XPS

## Abstract

Polyolefins are considered among the most difficult polymeric materials to treat because they have poor adhesive properties and high chemical barrier responses. In this paper, an in-depth study is reported for the low pressure plasma (LPP) treatment of neutral polypropylene to improve adhesion properties. Changes in wettability, chemical species, surface morphology and roughness of the polypropylene surfaces were evaluated by water contact angle measurement, X-ray photoelectron spectroscopy and, furthermore, atomic force microscopy (AFM). Finally, the bonded joints were subjected to tensile tests, in order to evaluate the practical effect of changes in adhesion properties. The results indicate that plasma is an effective treatment for the surface preparation of polypropylene for the creation of bonded joints: contact angles decreased significantly depending on the plasma-parameter setup, surface morphology was also found to vary with plasma power, exposure time and working gas.

## 1. Introduction

Thanks to the excellent macroscopic properties of polymeric materials, in recent years a noticeable increase has been observed in their use in industrial sectors characterized by medium-high technology levels. Some polymeric materials, such as polyolefins, in fact, manage to combine excellent properties of specific strength (lightness combined with good mechanical strength) with chemical inertia, easy processability, possibility of being colored and excellent surface finish [1,2]. All these factors promote the rising use of these materials in technological sectors such as medicine, automotive, aerospace, electronics, etc.

In general terms, polymer materials are characterized by high chemical inertness, which leads to very low surface energy values and, consequently, poor adhesive properties. On the other hand, hydrophilicity could be beneficial in a huge number of applications involving adhesive bonding, painting, labelling and traceability [3,4,5]. The only way to increase the hydrophilic properties of the surfaces is to carry out a surface treatment. Polyolefins are considered among the most difficult polymeric materials to treat because they have poor adhesive properties and high chemical barrier responses. Wettability and adhesion characteristics are important research topics, since these factors strongly affect the success of a surface treatment prior to bonding, for the short-term and long-term performance of joints. Wettability and surface energy of the substrates with respect to the adhesive are critical for the creation of secondary bonds in the adsorption theory. Therefore, the main objective of a surface treatment is to increase the surface energy of the substrate as much as possible [5,6,7,8].

Traditional methods mainly involve chemical treatments. ASTM D2093 [9] suggests a treatment with sulfuric acid-dichromate solution but does not suggest any physical treatment. R. Wegman added to this a bleach-detergent treatment and a Lead Dioxide-Sulfuric Acid Treatment [10]. The use of chemical treatments has given good results but, generally, these treatments generate great amounts of waste that must be appropriately managed and pose a risk for the operators. In recent years, even if they are not consolidated as standard procedures, methods based on the use of an electrical discharge through a gas (plasma treatments) have been extensively used as surface treatments [11]. They present great advantages: the possibility of treating the topmost surface layer without affecting bulk properties, flexibility in the treatment of different kinds of materials, non-heating of the samples and high environmental efficiency (no waste generation). Among the different existing plasma treatments (corona, atmospheric, etc.), low-pressure glow discharge plasma (commonly known as LPP), allows better control of the processing parameters, and this determines high consistency and reproducibility in results. As Encinas et al. highlighted in many studies [12], it produces a remarkable increase in adhesive properties acting on the surface by cleaning, etching, and introducing new functionalities. The main mechanisms involved are: surface grafting or breakdown of polymer chains and reaction of radical fragments with active species.

In a previous study, the same authors demonstrated an improvement in adhesion properties of plasma treated polyethylene in terms of wettability, evaluated by contact angle measurement, and lap shear strength of the adhesive bonded joints realized using treated surfaces. The results were compared with untreated substrates and a conventional primer treatment [13].

Sanchis et al. established that O_2_ plasma treatment on polyethylene films greatly enhances polar component to surface energy values which is revelatory that one of the main plasma mechanisms is surface activation due to the creation of polar groups (mainly =CO, –COOH and –OH groups). A minor increase in surface roughness due to surface etching was also revealed [14].

In their review on surface modification of polymers for biomedical application [15], Yoshida et al. presented several profound effects of plasma treatments: inserting functional groups, graft polymerization, crosslink formation, surface roughness control, and thin film coating to enhance biocompatibility and controlled drug release.

Based on several studies, many researchers commonly believe that the functional groups introduced on the surface react with the adhesive, forming chemical bonds at the polymer/adhesive interface. This results in an improved adhesive strength. However, the correlation between the number of functional groups and the adhesive strength has been discussed in very few studies. Therefore, it is of great concern to correlate the effect of LPP on the surface modification of polymeric substrates in terms of wettability, chemical changes and surface morphology, to the bonding mechanism between adhesive and substrate. The intent of this paper is to provide guidelines and quantitative indications for the modification of a polypropylene substrate. In fact, even if many works have been carried out on the surface modification of polymers, mainly by plasma, to the authors’ knowledge, no studies on statistical analysis, which correlates plasma parameters, surface characteristics and mechanical performance, have been conducted until now.

In order to investigate the effect of process working gas on the surface properties and correlate it to mechanical characteristics of adhesive-bonded lap-shear polypropylene samples, two LPP treatments using air and oxygen were applied. Contact angle measurements and surface free energy determination were performed to assess adherend wetting properties. Quasi-static tests were conducted to evaluate the effect of plasma treatment on the adhesive bond strength. Atomic force microscopy (AFM) was utilized to characterize the morphological modification induced by plasma treatments. Finally, an accurate X-ray photoelectron spectroscopy (XPS) survey on C1s peak was conducted to determine chemical-state on the plasma-treated polypropylene.

## 2. Materials and Methods

### 2.1. Materials and Surface Treatment

Neutral polypropylene (PP) samples, 2 mm-thick, were prepared for plasma. Samples of different dimensions were cut to size for various analyses. Samples of 10 × 10 × 2 mm were prepared for surface characterization, while rectangular specimens of dimensions 100 × 25 × 2 mm were used to make bonded joints, according to ASTM D3136 standard [16]. Table 1 reports the main mechanical characteristics of the substrate material.

A two-component epoxy adhesive, 3M™ DP490, was used to manufacture the adhesively bonded joints; the resin and hardener were mixed in a stoichiometric ratio of 2:1.

**Figure 1:**
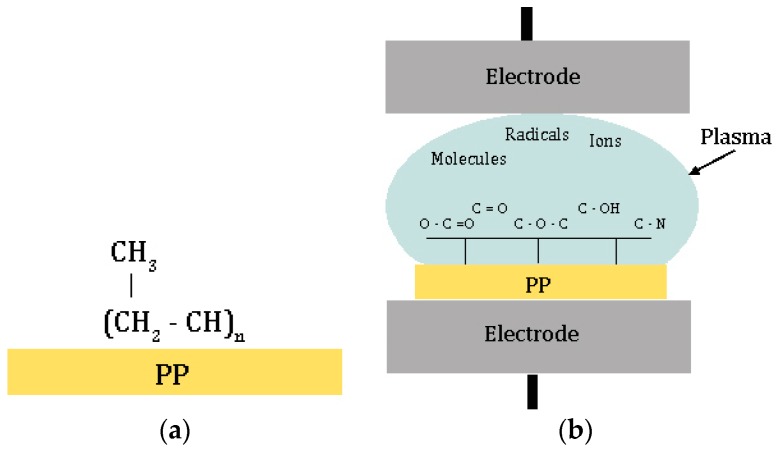
Scheme of the low-pressure glow discharge plasma (LPP) surface modification: (**a**) Polypropylene (PP) base composition, (**b**) possible polar groups introduced in the outer most layers of the surface by reactions with plasma species (mainly radicals, ions and molecules).

In order to evaluate the effect of plasma treatment on the adhesion characteristics of polypropylene, some surface preparations were carried out going to vary the main working parameters, i.e., power, time and process gas. A schematic representation of the plasma surface modification is reported in Figure 1 [11,17].

All the treatments were preceded by a degreasing of the surface with a cloth soaked in acetone. The substrates were then treated using a low pressure plasma. In particular, a radio frequency (RF) generator operating at 13.56 MHz was used (Gambetti Kenologia, Binasco, Italy). The chamber had a diameter of 150 × 330 mm in length and plasma acted on a total surface of 435 cm^2^. The process gases used were air and oxygen at a flow rate of 0.025 SLM. The base pressure was set to 0.1 mbar. On the basis of a previous study, for both process gases, three power levels (50, 125 and 200 W) and three exposure times (5, 180 and 300 s) were used. Solvent decrease samples (S) were employed as control.

### 2.2. Surface Characterization

The effect of plasma process parameter on surface modification was assessed through evaluation of the contact angle, survey on the surface chemical composition and an investigation of surface morphology and roughness. The contact angle formed on the substrates by the two probe liquids, namely deionized H_2_O and diiodomethane (CH_2_I_2_), whose characteristics are reported in Table 2, were evaluated, in order to calculate the polar and dispersive component of the surface energy, by Owens–Wendt regression model [18].

A Theta Lite optical tensiometer (Attension-Biolin Scientific, Gothenburg, Sweden) was used to measure the profiles of sessile droplets on PP surfaces. Ten droplets of constant volume (3 μL for H_2_O and 2 μL for CH_2_I_2_) were deposited on the substrate surface by means of a micro-syringe. The image was processed by using the affiliated software, OneAttension, which provided a view of the distension of the drop on the substrate and real time acquisition of the angle values.

X-ray photoelectron spectroscopy (XPS) was used to investigate the surface functionalization of the plasma modified PP substrates. In particular, an XPS Kratos Axis UltraDLD (Kratos Analytical Ltd., Manchester, UK) instrument was used to perform the analyses. It was equipped with a monochromatic Al Kα source (1486.6 eV), operating at 15 kV and 20 mA. The area actually subjected to the survey was 700 × 300 μm^2^. Through the software CasaXPS, spectra were acquired in survey mode, both at low and high resolution, over the entire range of available energies. All spectra were calibrated with reference to the peak C1s, which was centered at a binding energy (*Eb*) value equal to 284.8 eV (C–C bond). During data acquisition, a Kratos charge neutralization system was used. The surface investigation focused on the most significant samples, selected on the outcome of the wettability tests. The only-degreased sample was used as a reference to evaluate the surface chemical modifications brought about by LPP treatment.

To investigate the effect of treatment on the surface morphology modification, measurements were carried out using an atomic force microscope (AFM), an “MFP-3D” (Asylum Research, Goleta, CA, USA). Topographic images were acquired, by scanning spot areas over the surface using a probe consisting of a cantilever working in tapping-mode. The probe used in the measurements was an NCHR Nanosensor, whose main characteristics were:

- peak radius of curvature <10 nm;
- tip height between 10 and 15 μm;
- material: silicon doped with an aluminum layer, to increase the signal of reflectivity.

The roughness measurements of the specimens were repeated three times on a reference area of 90 × 90 μm at a scan rate of 0.15 Hz. Feedback signals were reprocessed using the “AR Igor Pro” software; the variation in the surface roughness of the plasma-treated and untreated substrates was expressed as an average quadratic variation of the vertical *z* dimension values in the areas examined, calculated using the following equation [19]:

(1) $$S_{q}=\sqrt{\frac{1}{A}}\iint^{}z^{2}\left(x,y\right)dx\;dy.$$

### 2.3. Adhesive-Joint Manufacturing and Lap-Shear Tests

The effect of the modification of the adhesion characteristics was evaluated by tensile shear tests using single-lap joints. The specimen geometry and test conditions followed the ASTM D3163 standard [16]. Adhesive was applied to the faying surface of each substrate, in order to obtain a bonded area of 12.5 × 25 mm. The specimens had two rectangular adherends of dimensions 25 × 100 × 2 mm. A comb polytetrafluoroethylene device was used to achieve the bondline thickness of 0.5 mm and to ensure proper alignment. The test was performed using an Instron 8802 test machine at a test speed of 1.3 mm/min. For each set of treatment conditions, five repetitions were performed, and the mean value was taken as shear strength. A simple statistical analysis, using Pearson’s coefficients [20,21], was used to understand the influence of the different plasma parameters. Furthermore, the same analysis was conducted in order to correlate the surface modification induced by plasma with the shear strength values, evaluated by lap-shear tests.

## 3. Results and Discussion

### 3.1. Surface Wettability

**Figure 2:**
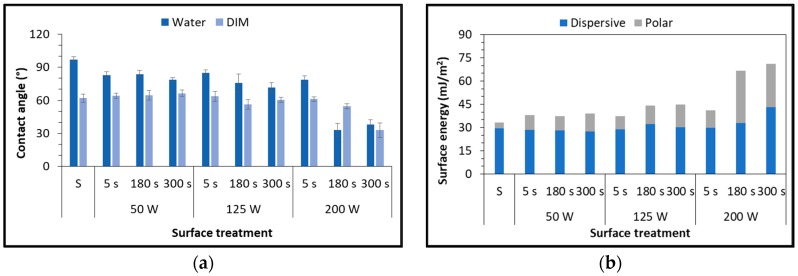
Static water and diiodomethane (DIM) contact angle (**a**) and surface free energy (**b**) of PP substrates as a function of plasma parameters, using air as working gas.

Surface treatment of the polypropylene with LPP gives very different results on contact angle evaluation varying the working gas. Figure 2a shows the dependence of the water contact angles on plasma treatment parameters, using air as working gas. The water contact angle of untreated substrate was 97.1°, which is in good agreement with [22]. In order to obtain significant results, it is necessary to increase the power input to 200 W and extend the exposure time to 180 s. DIM contact angle, corresponding to the dispersive component of surface free energy, has the same behavior, even though the decrease is much lower.

**Figure 3:**
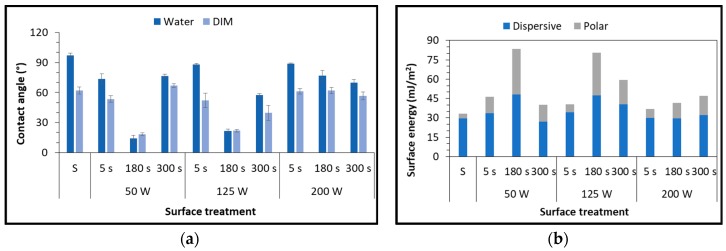
Static water contact angle (**a**) and surface free energy (**b**) of PP substrates as a function of plasma parameters, using oxygen as working gas.

The surface free energy, reported in Figure 2b as a function of plasma parameters, provides the explanation to the contact-angle observations: for high values of power input and exposure time the polar component rises and thus the wettability increases. On the contrary, a significant decrease in water contact angle could be reached using the same parameters but oxygen as working gas (Figure 3). The central values of exposure time (180 s) correspond to the best results (14° for the 50 W and 21° for the 125 W treatments), confirmed by a substantial increase in surface energy of the polymeric substrates.

The polar component (Figure 3b) of the 50 W-180 s and 125 W-180 s are the highest reached in this test campaign.

Previous studies established a relationship between the increase of surface energy and an increase in the polar component of the energy. Since it is related to the increase in the presence of oxygen-based functional groups [23,24], this results in a larger number of chemical bonds that can be built with the adhesive molecules. This work confirms studies performed by Morent et al. [25], who experimented the effect of a plasma dielectric barrier discharge (DBD) in medium pressure upon polypropylene films. In that case, the large reduction in contact angle was mainly due to formation of oxygen-containing functionalities, in particular C–O, O–C=O and C=O. Similar results were also obtained by the same authors on other polymeric substrates, such as PA6 and PA66 [26].

### 3.2. Surface Chemistry

XPS analysis was performed to extend the outcomes of the contact angle measurements, which suggests a surface oxidation after plasma treatment of PP substrates. In particular, the atomic composition of the untreated and the plasma-modified PP films was determined. As mentioned above, the surface investigation focused on the most significant samples, selected on the outcome of the previous tests. The only-degreased was used as a control sample to evaluate the surface chemical modifications brought about by LPP treatment. Table 3 reports the results obtained from the general survey, and two interesting relationships, i.e., O/C and N/C ratios. Presence of oxygen on the control surface suggests that PP contains some contaminations and/or more probably low grade surface oxidation [8].

The survey reveals that plasma modification results in a significant incorporation of oxygen, while only a very small increase in nitrogen content into the surface after plasma treatment was detected. This confirms that the reduction in contact angle values by exposure to both gas plasma treatments is mainly due to formation of oxygen-containing, and therefore polar, functional groups on the polypropylene surfaces.

**Figure 4:**
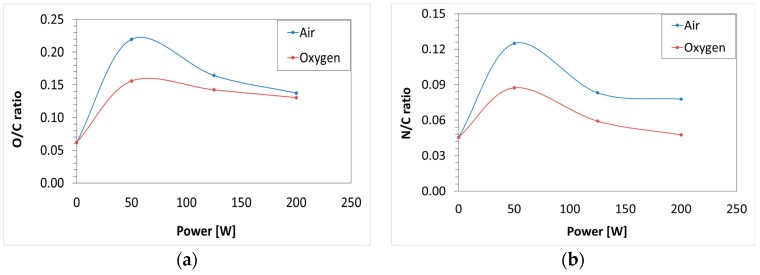
X-ray photoelectron spectroscopy (XPS) atomic ratios O/C (**a**) and N/C (**b**) of polypropylene surfaces with respect to plasma treatment parameters.

As could be immediately observed in Figure 4, the ratios increased for all the setup parameters, from a minimum value of about 110% to a maximum of 254% compared to the control sample. Lower power value of 50 W gave the highest increase for both working gases and considering both ratios.

**Figure 5:**
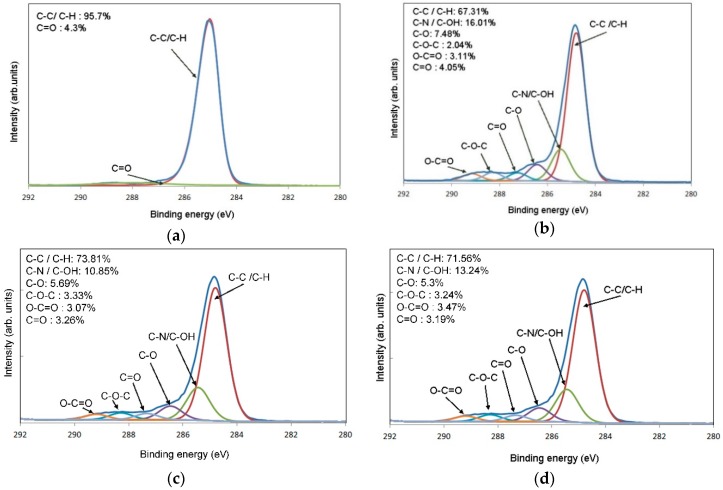
High resolution C1s spectra of (**a**) untreated and air treated samples with different power inputs: (**b**) 50 W, (**c**) 125 W and (**d**) 200 W.

In order to investigate the chemical groups introduced on the PP surface by plasma treatment, high-resolution spectra of the C1s peaks was investigated in detail. In this regard, Table 3 also presents the results of the C1s peak deconvolution which indicate the distinct difference in concentration of polar functional groups on the PP substrates after plasma treatment. The components of the C1s peak are indicated in Figure 5, where the case of air treatments are reported as an example. Such components were attributed to C–C or C–H, C–N or C–OH, C–O, C–O–C, O–C=O and C=O, according to known literature values [27].

### 3.3. Surface Morphology

**Figure 6:**
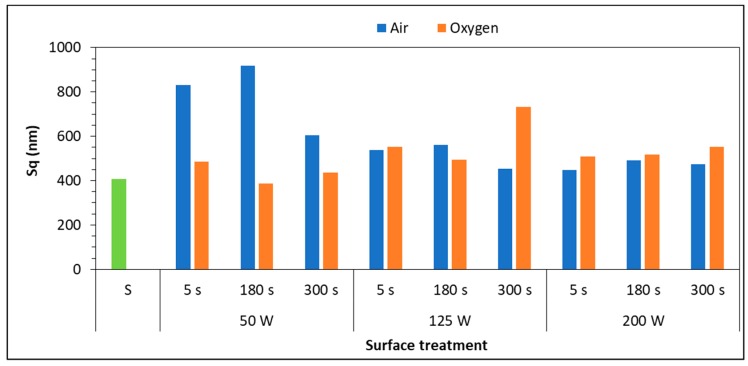
Surface roughness of PP substrates with respect to plasma treatment parameters.

Plasma could affect surface morphology and therefore surface roughness, especially on the nanoscale, attributed to the impact of ions generated during plasma process. The parameter selected to describe the surface roughness characteristics was the mean square roughness S_q_, because it is particularly more sensitive to high peaks and deep morphological valleys. The research was carried out on the samples treated with the different process parameters with only degreased substrates as control. Figure 6 shows surface roughness of polypropylene substrates treated with different gases.

**Figure 7:**
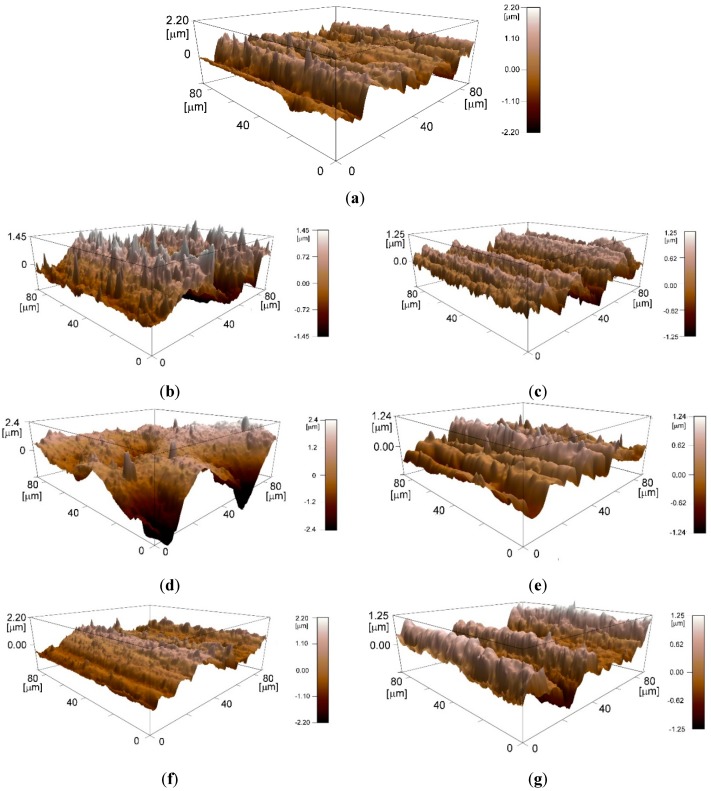
Atomic force microscope (AFM) 3D-topographic images of untreated (**a**) and plasma treated PP substrates, in particular: (**b**) Air treatment 50 W-180 s; (**c**) air treatment 125 W-180 s; (**d**) air treatment 200 W-180 s; (**e**) oxygen treatment 50 W-180 s; (**f**) oxygen treatment 125 W-180 s; (**g**) oxygen treatment 200 W-180 s.

Previous studies [23,28,29,30,31,32] assessed the effect of surface roughness on the bonding strength of the adhesive-bonded joints but no convergence on the correlation between surface texture, surface energy and adhesive joint strength was found, at least for polymeric substrates. This is mainly due to the fact that in adhesive bonding technology the different parts of the adhesive system, (i.e., substrate, interface, adhesive) are strongly related one to each other and all concur to the performance of the bonded joint. From the AFM results described above, it was found that only 50 W treatment, performed with air and treatment times of 5 and 180 s could create appreciable changes on morphology and roughness of the PP surfaces. Figure 6 reports the different morphologies created on the surface, varying the working gas and the power input, compared to the control sample (Figure 7a).

Compared to the other air treatments, the longest one creates a smooth surface (Figure 7d), as a sort of too intense shot peening.

Morphology created by oxygen is very similar to untreated ones, for all the power inputs, and this is in agreement with the numerical results reported in Figure 6. Hnilica et al. [28] identified similar behavior on PA12 treated with a microwave plasma jet: the effects induced by the oxygen treatment were mostly chemical and not morphological.

### 3.4. Lap-Shear Strength

**Figure 8:**
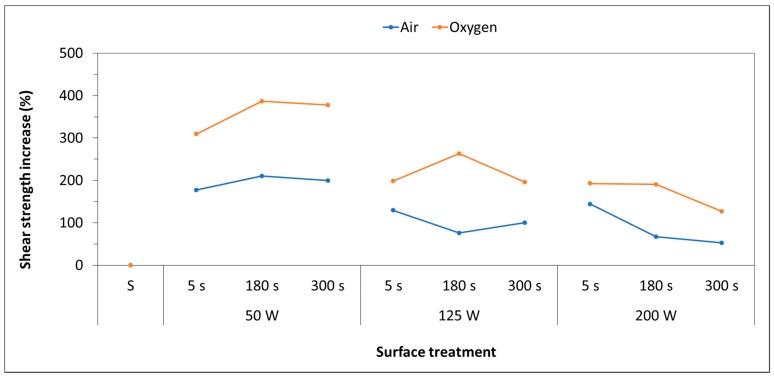
Shear strength increase of adhesive bonded joints as a function of LPP process parameters.

To evaluate the efficacy of the LPP on the increase of adhesive properties, plasma-treated polypropylene samples were joined by using an epoxy adhesive and mechanical performance of the adhesion joints was tested using lap-shear test. Figure 8 shows plots of the shear strength increase as a function of the treatment parameters for the different process gases.

As shown, the strength of the adhesive-bonded treated polypropylene is significantly higher than that of the adhesive-bonded untreated one. In fact, compared to the control specimens, plasma treated ones demonstrated a statistically significant increase of shear strength, from a minimum of 52.5% to a maximum of 386.6%. For each working gas, a significant increase in shear strength was obtained using 50 W of power input, for any treatment duration. In the opposite case (maximum power input 200 W), the lowest increases are obtained, but even in this case, shear strength is higher than the values obtained with untreated substrates. Hence, even in these conditions, the effectiveness of the LPP treatment is proven.

A statistical analysis, using Pearson’s coefficients (Table 4), underlines that for both working gases the most influential parameter on the shear strength is the power input. In particular, there is an inverse proportionality between the power input and the shear strength of the bonded joints.

**Figure 9:**
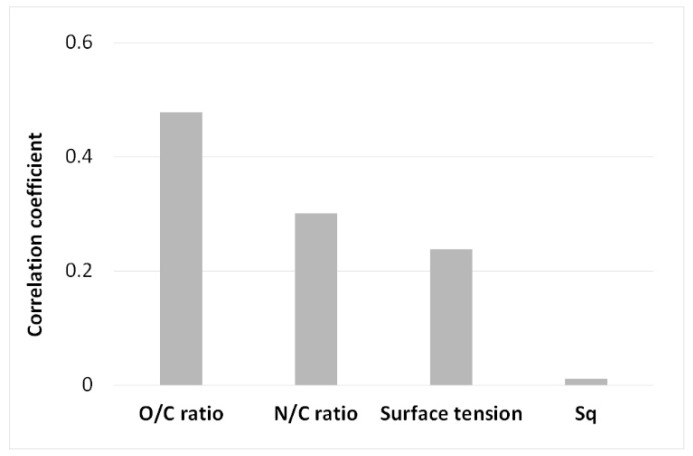
Graph of the correlation strength between the shear strength and the different surface characteristics.

Further analysis was than conducted in order to statistically correlate the surface modification induced by plasma and evaluated in the previous paragraphs, with the shear strength values. The correlation strength between the shear strength as a response and the main surface characteristics was calculated using the Pearson correlation coefficient, reported in Figure 9. The chemical insertion of polar species is most notably directly proportional, resulting in a great effect on the mechanical characteristics of adhesively bonded joints. Moreover, slower correlation strength was exhibited for both the N/C ratio and the surface tension, while approximately no correlation was noted with S_q_. This aspect implies that for this specific adhesive system, made up of polymer substrates, the insertion of polar species creates the optimal interface condition with epoxy adhesive, irrespective of the surface morphology that plasma had not substantially modified.

Only little bibliography reports lap-shear tests performed on polymeric substrates (and not films), especially on polypropylene, but in any case, these results confirm the expected use of different plasma sources. For example, Encinas et al. obtained similar results on polypropylene, both in terms of shear strength improvement and failure mode using an atmospheric plasma (APPT): lap-shear strength on adhesive bonded joints revealed an important enhancement of tensile strength of about 500%, when the APPT treatment was employed [33]. The study by Pandiyaraj et al. on polypropylene films confirm the expected use of a vacuum plasma to increase T-peel and lap-shear strength [23,34]. Furthermore, the functionalization of polymeric substrates is often reported as an advantage in terms of shear and T-peel strength increase [29,35].

## 4. Conclusions

To understand the relationship between the surface properties and adhesive bond performance, 2 mm-thick polypropylene substrates were plasma treated using an LPP system and optimizing the working parameters. Extensive tests conducted on assessing the effect of plasma gas on contact angle, surface free energy, chemical and morphological modifications and strength of the adhesive-bonded joints resulted in the following:

- Plasma treatments significantly decreased the water contact angle of the polymer surfaces, especially pronounced for specific parameter sets. Consequently, LPP increased the surface free energy of PP compared to that of untreated samples, mainly thanks to an increase of the polar component rather than the dispersive one.
- Oxygen-containing functional groups were formed on the treated surfaces due to ionization of both working gases, which are the principal cause of the improved wettability.
- The analysis of the treated samples using AFM revealed that changes in surface morphology using LPP treatments are considerable only for specific sets-up;
- The main surface modifications, which affect mechanical characteristics of bonded joints, are the insertion of polar species on the polypropylene substrate surfaces.

## Figures and Tables

**Table 1:** Mechanical and thermal properties of the substrates used for the tests.

Mechanical Properties	Value
Yield stress (MPa)	25
Elongation at break (%)	6
Tensile modulus of elasticity (MPa)	1300
Charpy impact strength (kJ/m^2^)	13
Rockwell hardness (R scale)	85

**Table 2:** Dispersive, polar and total components of the two liquids.

Liquid	$\gamma_{lg}^{d}\;[\mathrm{mN}/m]$	$\gamma_{lg}^{p}\;[\mathrm{mN}/m]$	$\gamma_{sg}\;[\mathrm{mN}/m]$
Water	21.8	51	72.8
Diiodomethane (DIM)	50.8	0	51

**Table 3:** Surface elemental composition and elemental ratio of the plasma-treated polypropylene substrate for different process parameters.

Treatment			Concentration [at. %]			Relationships		Contribution of C1s components (%)					
Power Input	Time	Gas	O1s	C1s	N1s	O/C ratio	N/C ratio	C–C C–H	C–N C–OH	C–O	C–O–C	O–C=O	C=O
0	0	0	5.70	91.96	0.26	0.062	0.046	95.7	-	-	-	-	4.3
50	180	Air	16.25	73.98	2.03	0.220	0.125	67.31	16.01	7.48	2.04	3.11	4.05
50		Oxygen	12.93	82.94	1.13	0.156	0.087	67.1	16.48	6.98	3.21	2.75	3.48
125	180	Air	13.12	79.78	1.09	0.164	0.083	73.81	10.85	5.69	3.33	3.07	3.26
125		Oxygen	12.00	84.24	0.71	0.142	0.059	67.45	14.44	7.39	3.65	3.24	3.83
200	180	Air	11.44	83.22	0.89	0.137	0.078	71.56	13.24	5.3	3.24	3.47	3.19
200		Oxygen	11.12	85.17	0.53	0.131	0.048	67.18	15.57	7.76	3.82	2.56	3.11

**Table 4:** Pearson’s coefficients.

Parameter	Working Gas	
	Air	Oxygen
**Power input**	−0.798	−0.894
**Time**	−0.257	0.028

## References

[1] Agarwal S., Arisman R.K., Baghdachi J., Benson R., Belcher S.L., Berry M., Cantor K.M., Carroll W.F., Coleman E.A., Coutinho C. (2011). Applied Plastics Engineering Handbook.

[2] Platt D.K. (2003). Industrial Applications for Engineering and High Performance Plastics. Engineering and High Performance Plastics-Market Report.

[3] Jofre-Reche J.A., Pulpytel J., Fakhouri H., Arefi-Khonsari F., Martín-Martínez J.M. (2016). Surface treatment of polydimethylsiloxane (PDMS) with atmospheric pressure rotating plasma jet. Modeling and optimization of the surface treatment conditions. Plasma Process. Polym..

[4] Belmonte G.K., Charles G., Strumia M.C., Weibel D.E. (2016). Permanent hydrophilic modification of polypropylene and poly(vinyl alcohol) films by vacuum ultraviolet radiation. Appl. Surf. Sci..

[5] Juang R.S., Hou W.T., Huang Y.C., Tseng Y.C., Huang C. (2016). Surface hydrophilic modifications on polypropylene membranes by remote methane/oxygen mixture plasma discharges. J. Taiwan Inst. Chem. Eng..

[6] Packham D.E. (2003). Surface energy, surface topography and adhesion. Int. J. Adhes. Adhes..

[7] Petasch W., Rauchle E., Walker M., Elsner P. (1995). Improvement of the adhesion of low-energy polymers by a short-time plasma treatment. Surf. Coat. Technol..

[8] Encinas N., Díaz-Benito B., Abenojar J., Martínez M.A. (2010). Extreme durability of wettability changes on polyolefin surfaces by atmospheric pressure plasma torch. Surf. Coat. Technol..

[9] ASTM International (2003). ASTM D2093-03. Standard Practice for Preparation of Surfaces of Plastics Prior to Adhesive.

[10] Wegman R.F. (2006). Surface Preparation Techniques for Adhesive Bonding.

[11] Hetemi D., Pinson J. (2017). Surface functionalisation of polymers. Chem. Soc. Rev..

[12] Encinas N., Dillingham R.G., Oakley B.R., Abenojar J., Martínez M.A., Pantoja M. (2012). Atmospheric pressure plasma hydrophilic modification of a silicone surface. J. Adhes..

[13] Mandolfino C., Lertora E., Gambaro C., Bruno M. (2014). Improving adhesion performance of polyethylene surfaces by cold plasma treatment. Meccanica.

[14] Sanchis M.R., Blanes V., Blanes M., Garcia D., Balart R. (2006). Surface modification of low density polyethylene (LDPE) film by low pressure O2 plasma treatment. Eur. Polym. J..

[15] Yoshida S., Hagiwara K., Hasebe T., Hotta a. (2013). Surface modification of polymers by plasma treatments for the enhancement of biocompatibility and controlled drug release. Surf. Coatings Technol..

[16] ASTM International (2001). ASTM D3163-01. Standard Test Method for Strength Properties of Adhesively Bonded Plastic Lap-Shear Joints in Shear by Tension Loading.

[17] Thakur V.K., Vennerberg D., Kessler M.R. (2014). Green aqueous surface modification of polypropylene for novel polymer nanocomposites. ACS Appl. Mater. Interfaces.

[18] Owens D.K., Wendt R.C. (1969). Estimation of the surface free energy of polymers. J. Appl. Polym. Sci..

[19] Griffiths B. (2001). Surface Finish Characterization. Manufacturing Surface Technology: Surface Integrity and Functional Performance.

[20] Kirch W. (2008). Pearson’s Correlation Coefficient. Encyclopedia of Public Health.

[21] Chang Y., Yang D., Guo Y. (2018). Laser ultrasonic damage detection in coating-substrate structure via Pearson correlation coefficient. Surf. Coat. Technol..

[22] Chen W.X., Yu J.S., Hu W., Chen G.L. (2016). Partial hydrophilic modification of biaxially oriented polypropylene film by an atmospheric pressure plasma jet with the allylamine monomer. Appl. Surf. Sci..

[23] Pandiyaraj K.N., Selvarajan V., Deshmukh R.R., Gao C. (2009). Modification of surface properties of polypropylene (PP) film using DC glow discharge air plasma. Appl. Surf. Sci..

[24] Chen Y., Gao Q., Wan H., Yi J., Wei Y., Liu P. (2013). Surface modification and biocompatible improvement of polystyrene film by Ar, O2 and Ar+O2 plasma. Appl. Surf. Sci..

[25] Morent R., De Geyter N., Leys C., Gengembre L., Payen E. (2008). Comparison between XPS- And FTIR-analysis of plasma-treated polypropylene film surfaces. Surf. Interface Anal..

[26] Mandolfino C., Lertora E., Gambaro C. (2017). Influence of cold plasma treatment parameters on the mechanical properties of polyamide homogeneous bonded joints. Surf. Coat. Technol..

[27] Van der Heide P. (2011). X-ray Photoelectron Spectroscopy: An Introduction to Principles and Practices.

[28] Hnilica J., Potočňáková L., Stupavská M., Kudrle V. (2014). Rapid surface treatment of polyamide 12 by microwave plasma jet. Appl. Surf. Sci..

[29] Fombuena V., Balart J., Boronat T., Sánchez-Nácher L., Garcia-Sanoguera D. (2013). Improving mechanical performance of thermoplastic adhesion joints by atmospheric plasma. Mater. Des..

[30] Slepic P., Bla O., Kota V., Sajdl P., Hnatowicz V. (2006). Modification of surface properties of polyethylene by Ar plasma discharge. Nucl. Instrum. Methods Phys. Res. B.

[31] Gao Z., Sun J., Peng S., Yao L., Qiu Y. (2009). Surface modification of a polyamide 6 film by He/CF4 plasma using atmospheric pressure plasma jet. Appl. Surf. Sci..

[32] Baldan A. (2004). Adhesively-bonded joints and repairs in metallic alloys, polymers and composite materials: Adhesives, adhesion theories and surface pretreatment. J. Mater. Sci..

[33] Encinas N., Abenojar J., Martinez M. (2012). Development of improved polypropylene adhesive bonding by abrasion and atmospheric plasma surface modifications. Int. J. Adhes. Adhes..

[34] Navaneetha Pandiyaraj K., Selvarajan V., Deshmukh R.R., Gao C. (2008). Adhesive properties of polypropylene (PP) and polyethylene terephthalate (PET) film surfaces treated by DC glow discharge plasma. Vacuum.

[35] Oosterom R., Ahmed T.J., Poulis J.A., Bersee H.E.N. (2006). Adhesion performance of UHMWPE after different surface modification techniques. Med. Eng. Phys..

